# Sublingual Ketamine for Treatment Resistant Depression: Embracing the Change

**DOI:** 10.1192/bjo.2025.10211

**Published:** 2025-06-20

**Authors:** Ranjini Rao

**Affiliations:** The Geelong Clinic, Geelong, Australia

## Abstract

**Aims:** 1. Examine efficacy and durability of antidepressant effects. 2. Identify specific symptoms of improvement. 3. Compare response rates and duration of antidepressant effects in failed to respond to previous treatments.

**Methods:** Prospective review study of patients who received off-label rapid dissolve wafer for SL delivery in hospital setting 2–3 times a week for 4 weeks. The study examined the effectiveness and safety of the clinical use on depression and anxiety symptoms. The regimen was determined by Psychiatrist experiential CME in ketamine therapy. Dosing increases were made based on patient’s report, clinical response and side effects. Optimisation of maintenance dose based on pharmacokinetic modelling to achieve serum (2–2.5 mg/kg wt.). Clinician rated scales MADRS, MoCA and CADSS were completed to evaluate response & KSET protocol for side effects. Patient completed the online experience survey within 24 hours of dose administration.

**Results:** N=60 with no dropouts from treatment and no patients were hospitalized during treatment course. The mean patient age was 45.4, most common comorbid psychiatric diagnoses were Generalized Anxiety Disorder and mean of 3.6 previous psychotropic drug trials. Responders had > dissociation (mean CADSS 11.8). We found a moderately strong but not statistically significant correlation between the CADSS score during treatment and the reduction in MADRS from baseline to week 4 (r=0.52, p=0.059). Minor adverse events and side effects including nausea, dizziness, headache, loss of balance, were assessed and were self-limited and resolved without medical intervention.

Effectiveness – nearly 50% of patients with moderate to severe depression saw an improvement of MADRS and GAD-7 scores. This reduction rate improved to 60% in patients who completed a clinically recommended 12 ketamine course.

**Conclusion:** Increased dissociation correlated moderately strongly, but not significantly, with a decrease in depressive symptoms. Reductions in scores matched those with 6 vs 12 completed treatments and it is reasonable to speculate that there would be maintenance of improvement with additional treatments.

